# New Eugenol Derivatives with Enhanced Insecticidal Activity

**DOI:** 10.3390/ijms21239257

**Published:** 2020-12-04

**Authors:** Maria José G. Fernandes, Renato B. Pereira, David M. Pereira, A. Gil Fortes, Elisabete M. S. Castanheira, M. Sameiro T. Gonçalves

**Affiliations:** 1 Centre of Chemistry, Department of Chemistry, Campus of Gualtar, University of Minho, 4710-057 Braga, Portugal; mjfernandes@quimica.uminho.pt (M.J.G.F.); gilf@quimica.uminho.pt (A.G.F.); 2 REQUIMTE/LAQV, Laboratory of Pharmacognosy, Department of Chemistry, Faculty of Pharmacy, University of Porto, R. Jorge Viterbo Ferreira, 228, 4050-313 Porto, Portugal; rjpereira@ff.up.pt; 3 Centre of Physics, Department of Physics, Campus of Gualtar, University of Minho, 4710-057 Braga, Portugal; ecoutinho@fisica.uminho.pt

**Keywords:** eugenol derivatives, semisynthetic insecticides, phenylpropanoids, *Spodoptera frugiperda*, natural product-derived insecticides

## Abstract

Eugenol, the generic name of 4-allyl-2-methoxyphenol, is the major component of clove essential oil, and has demonstrated relevant biological potential with well-known antimicrobial and antioxidant actions. New *O*-alkylated eugenol derivatives, bearing a propyl chain with terminals like hydrogen, hydroxyl, ester, chlorine, and carboxylic acid, were synthesized in the present work. These compounds were later subjected to epoxidation conditions to give the corresponding oxiranes. All derivatives were evaluated against their effect upon the viability of insect cell line *Sf9* (*Spodoptera frugiperda*), demonstrating that structural changes elicit marked effects in terms of potency. In addition, the most promising molecules were evaluated for their impact in cell morphology, caspase-like activity, and potential toxicity towards human cells. Some molecules stood out in terms of toxicity towards insect cells, with morphological assessment of treated cells showing chromatin condensation and fragmentation, which are compatible with the occurrence of programmed cell death, later confirmed by evaluation of caspase-like activity. These findings point out the potential use of eugenol derivatives as semisynthetic insecticides from plant natural products.

## 1. Introduction

Due to the exponential increase in population, it is necessary to ensure that agricultural production follows the resulting food needs. The need for the prevention and control of plant diseases, as well as insect pests, is a crucial issue facing crop protection. To date, the most common strategy for controlling these issues has depended on the use of conventional pesticides, most of which are synthetic pesticides, including insecticides [1,2,3].

The intensive use of synthetic pesticides has resulted in damage to the environment, health hazards, and loss of biodiversity [4,5], so it is necessary to adopt less harmful strategies that can include the use of natural-based pesticides, which will result in a healthy environment and sustainable agriculture [6,7]. The concomitant use of natural pesticides/semisynthetic pesticides and synthetic pesticides could also take place as a changeover alternative to circumvent several negative effects of the exclusive use of synthetic compounds [8,9,10]. Owing to the structural diversity and biological activities of natural products, they could be rich sources of inspiration for the design optimization of active principles in formulation development [11,12].

Plants offer an extraordinary diversity of secondary metabolites, with proven efficacy against mosquito species of medical and veterinary importance, as well as against other noxious arthropod pests and vectors [13,14]. In recent years, the application of essential oils (EOs) and their bioactive compounds is gearing up rapidly as biopesticides, in order to limit the use of hazardous synthetic products, and these EOs are well-established as an alternative for the control of pre- and postharvest pests affecting agriculture-based food commodities [13,15,16]. These compounds have revealed great promise in applications like mosquito ovicide oils, given that they have shown particular potential as insecticides in organic agriculture [2,17].

In fact, EOs extracted by steam distillation of many aromatic plants have recently received a lot of attention due to their broad spectrum of action. These natural ingredients, composed of complex mixtures of monoterpenes, biogenetically related phenols, and sesquiterpenes [17,18,19], display antibacterial, antiviral, and antifungal activities, in addition to insecticide properties, as already mentioned [19,20,21,22,23,24]. Owing to their wide spectrum of activity, these compounds are nowadays considered as an alternative to chemicals in many applications, such as food preservation, pharmaceuticals, alternative medicine, and natural therapies [25,26,27]. EOs are typically characterized by a low melting point; most of them are liquid at room temperature, and their application in plant protection has some limitations, due to their poor solubility in water and high volatility. However, they display efficacy, biodegradability, various modes of action, and low toxicity, as well as an availability of source materials [6].

Eugenol, the major component of *Syzygium aromaticum* (clove) oil, has been used as a starting material and building block molecule for the manufacturing of bioactive compounds, on account of its particular structure and ready availability, in addition to numerous applications found in pharmaceutical, food, agricultural, and cosmetics industries [28,29]. Eugenol has also demonstrated antimicrobial and antioxidant activities [30], being also a powerful insecticide, effective on a wide variety of domestic arthropod pests [31,32,33]. A structural modification of EOs has been shown to enhance the biocidal effect of these phytochemicals by increasing their activity [34,35].

Considering all the above facts, in the present work semisynthetic eugenol derivatives—namely, *O*-alkylated bearing the propyl chain with hydrogen, hydroxyl, ester, chlorine and carboxylic acid as terminals, as well as the corresponding *O*-alkylated oxiranes—were synthesized. The main objective behind obtaining these eugenol derivatives was their evaluation as possible semisynthetic insecticides. Therefore, the biological activity of all compounds compared to a commercial synthetic insecticide was tested against *Sf9* (*Spodoptera frugiperda*) insect cell line. The results turned out to be very promising for future applications as active ingredients in formulations, with structural changes eliciting marked effects in terms of potency and, equally important, low toxicity towards human cells.

## 2. Results

Eugenol **1** is easily obtained by hydrodistillation from clove, and is known for its various biological activities, as mentioned above, namely insecticidal. As an attempt to find semisynthetic alternatives with improved insecticidal activity, eugenol derivatives **2a–f** and **3a–e** were prepared; two of these have been shown to be highly and selectively toxic to insects, but not to human cells.

### 2.1. Synthesis of Eugenol Derivatives ***2a*–*f*** and ***3a*–*e***

**Scheme 1:**
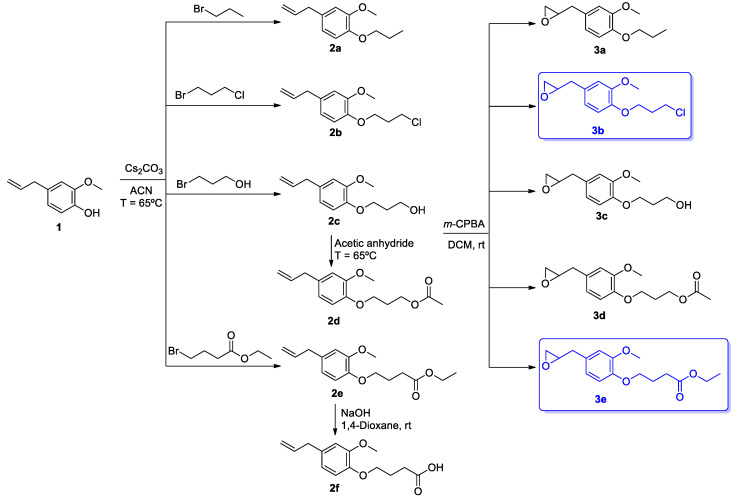
Synthesis of eugenol derivatives **2a–f** and **3a–e**.

4-Allyl-2-methoxyphenol, eugenol **1** was obtained by hydrodistillation from clove to a high degree of purity (≥95%), as confirmed by its ^1^H NMR spectrum, and used in the synthesis of six *O*-alkylated derivatives (**2a–f**) and five oxiranes (**3a–e**), as shown in Scheme 1. The structures of all the compounds were confirmed by ^1^H and ^13^C NMR spectroscopy and high-resolution mass spectrometry (HRMS), and the corresponding analytical data are shown below.

#### 2.1.1. 4-Allyl-2-Methoxyphenol **1**

Compound **1** was an off-white oil (14% yield of extraction).

^1^H NMR (CDCl_3_, 400 MHz): *δ*_H_ = 3.34 (2H, d, *J* = 6.8 Hz, CH_2_Ph), 3.89 (3H, s, OCH_3_), 5.07–5.12 (2H, m, CH=CH_2_), 5.81 (1H, broad s, OH), 5.93–6.03 (1H, m, CH=CH_2_), 6.70–6.88 (2H, m, H-3 and H-5), 6.87 (1H, d, *J* = 8.4 Hz, H-6) ppm.

#### 2.1.2. 4-Allyl-2-Methoxy-1-Propoxybenzene **2a**

Starting from compound **1** (0.200 g, 1.22× 10^−3^ mol) and using 1-bromopropane (0.12 mL, 1.34 × 10^−3^ mol), compound **2a** was obtained as a light yellow oil (0.224 g, 89% yield). *R*_f_ = 0.67 (ethyl acetate/light petroleum = 1:10). 1H NMR (CDCl_3_, 400 MHz): *δ*_H_ = 1.06 (3H, t, *J* = 7.6 Hz, OCH_2_CH_2_CH_3_), 1.88 (2H, sext, *J* = 7.2 Hz, OCH_2_CH_2_CH_3_), 3.35 (2H, d, *J* = 6.8 Hz, CH_2_Ph), 3.87 (3H, s, OCH_3_), 3.97 (2H, t, *J* = 7.2 Hz, OCH_2_CH_2_CH_3_), 5.07–5.14 (2H, m, CH=CH_2_), 5.94–6.04 (1H, m, CH=CH2), 6.72–6.74 (2H, m, H-3 and H-5), 6.83 (1H, d, *J* = 8.0 Hz, H-6) ppm. ^13^C NMR (CDCl_3_, 100.6 MHz): *δ*_C_ = 10.23 (OCH_2_CH_2_CH_3_), 22.36 (OCH_2_CH_2_CH_3_), 39.60 (CH_2_Ph), 55.69 (OCH_3_), 70.44 (OCH_2_CH_2_CH_3_), 112.24 (C-3), 113.06 (C-6), 115.30 (CH=CH_2_), 120.25 (C-5), 132.44 (C-4), 137.51 (CH=CH_2_), 146.76 (C-1), 149.23 (C-2) ppm. HRMS: *m/z* (ESI) calculated for C_13_H_19_O_2_ [M+1]^+^ = 207.1380; found = 207.1381.

#### 2.1.3. 4-Allyl-1-(3-Chloropropoxy)-2-Methoxybenzene **2b**

Starting from compound **1** (0.200 g, 1.22 × 10^−3^ mol) and using 1-bromo-3-chloro-propane (0.93 mL, 9.44 × 10^−3^ mol), compound **2b** was obtained as a light yellow oil (0.223 g, 72% yield). *R*_f_ = 0.67 (ethyl acetate/light petroleum = 1:10). ^1^H NMR (CDCl_3_, 400 MHz): *δ*_H_ = 2.28 (2H, quint, *J* = 6.0 Hz, OCH_2_CH_2_CH_2_Cl), 3.35 (2H, d, *J* = 6.8 Hz, CH_2_Ph), 3.78 (2H, t, *J* = 6.4 Hz, OCH_2_CH_2_CH_2_Cl), 3.86 (3H, s, OCH_3_), 4.15 (2H, t, *J* = 5.6 Hz, OCH_2_CH_2_CH_2_Cl), 5.06–5.13 (2H, m, CH=CH_2_), 5.93–6.03 (1H, m, CH=CH_2_), 6.72–6.74 (2H, m, H-3 and H-5), 6.86 (1H, d, *J* = 6.8 Hz, H-6) ppm. ^13^C NMR (CDCl_3_, 100.6 MHz): *δ*_C_ = 32.31 (OCH_2_CH_2_CH_2_Cl), 39.72 (CH_2_Ph), 41.64 (OCH_2_CH_2_CH_2_Cl), 55.85 (OCH_3_), 65.88 (OCH_2_CH_2_CH_2_Cl), 112.45 (C-6), 113.91 (C-3), 115.60 (CH=CH_2_), 120.48 (C-5), 133.37 (C-4), 137.55 (CH=CH_2_), 146.48 (C-1), 149.52 (C-2) ppm. HRMS: *m/z* (ESI) calculated for C_13_H_17_^35^ClNaO_2_ [M+Na = 263.0809; found = 263.0805; calculated for C_13_H_17_^37^ClNaO_2_ [M+Na]^+^ = 265.0783; found = 265.0780.

#### 2.1.4. 3-(4-Allyl-2-Methoxyphenoxy)Propan-1-ol **2c**

Starting from compound **1** (0.250 g, 1.52 × 10^−3^ mol) and using 3-bromopropan-1-ol (0.06 mL, 1.67 × 10^−3^ mol), compound **2c** was obtained as a light yellow oil (0.180 g, 53% yield). *R*_f_ = 0.23 (ethyl acetate/light petroleum 1:3). ^1^H NMR (CDCl_3_, 400 MHz): *δ*_H_ = 2.07 (2H, quint, *J =* 5.6 Hz, OCH_2_CH_2_CH_2_OH), 3.34 (2H, d, *J =* 6.4 Hz, CH_2_Ph), 3.85 (3H, s, OCH_3_), 3.88 (2H, t, *J =* 6.4 Hz, OCH_2_CH_2_CH_2_OH), 4.18 (2H, t, *J =* 5.6 Hz, OCH_2_CH_2_CH_2_OH), 5.05–5.12 (2H, m, CH=CH_2_), 5.91–6.01 (1H, m, CH=CH_2_), 6.71–6.73 (2H, m, H-3 and H-5), 6.84 (1H, d, *J =* 8.4, H-6) ppm. ^13^C NMR (CDCl_3_, 100.6 MHz): *δ*_C_ = 31.75 (OCH_2_CH_2_CH_2_OH), 39.82 (CH_2_Ph), 55.75 (OCH_3_), 61.56 (OCH_2_CH_2_CH_2_OH), 68.69 (OCH_2_CH_2_CH_2_OH), 112.06 (C-3), 113.48 (C-6), 115.64 (CH = CH_2_), 120.44 (C-5), 133.41 (C-4), 137.57 (CH=CH_2_), 146.46 (C-1), 149.37 (C-2) ppm. HRMS: *m*/*z* (ESI) calculated for C_13_H_19_O_3_ [M+1]^+^ = 223.1329; found = 223.1329.

#### 2.1.5. Ethyl 4-(4-Allyl-2-Methoxyphenoxy)Butanoate **2e**

Starting from compound **1** (0.200 g, 1.22 × 10^−3^ mol) and using ethyl 4-bromobutyrate (0.19 mL, 1.34 × 10^−3^ mol), compound **2e** was obtained as a colorless oil (0.249 g, 73% yield). *R*_f_ = 0.44 (ethyl acetate/light petroleum 1:10). ^1^H NMR (CDCl_3_, 400 MHz): *δ*_H_ = 1.24 (3H, t, *J =* 8.4 Hz, CO_2_CH_2_CH_3_), 2.14 (2H, quint, *J =* 6.4 Hz, OCH_2_CH_2_CH_2_CO_2_CH_2_CH_3_), 2.53 (2H, t, *J =* 7.2 Hz, OCH_2_CH_2_C*H_2_*CO_2_CH_2_CH_3_), 3.33 (2H, d, *J =* 6.8 Hz, CH_2_Ph), 3.85 (3H, s, OC*H_3_*), 4.04 (2H, t, *J =* 6.4 Hz, OC*H_2_*CH_2_CH_2_CO_2_CH_2_CH_3_), 4.14 (2H, q, *J =* 7.2 Hz, OCH_2_CH_2_CH_2_CO_2_C*H_2_*CH_3_), 5.04–5.11 (2H, m, CH=CH_2_), 5.91–6.01 (1H, m, CH=CH_2_) 6.69–6.72 (2H, H-3 and H-5), 6.82 (1H, d, *J =* 7.2, H-6) ppm. ^13^C NMR (CDCl_3_, 100.6 MHz): *δ*_C_ = 14.14 (OCH_2_CH_2_CH_2_CO_2_CH_2_*C*H_3_), 24.57 (OCH_2_*C*H_2_CH_2_CO_2_CH_2_CH_3_), 30.73 (OCH_2_CH_2_*C*H_2_CO_2_CH_2_CH_3_), 39.74 (*C*H_2_Ph), 55.85 (O*C*H_3_), 60.31 (OCH_2_CH_2_CH_2_CO_2_*C*H_2_CH_3_), 68.09 (CO*C*H_2_CH_2_CH_2_CO_2_CH_2_CH_3_), 112.41 (C-3) 113.60 (C-6), 115.54 (CH = *C*H_2_), 120.44 (C-5), 133.06 (C-4), 137.60 (*C*H = CH_2_), 146.58 (C-1), 149.46 (C-2), 173.20 (*C*O_2_CH_2_CH_3_) ppm. HRMS: *m/z* (ESI) calculated for C_16_H_23_O_4_ [M+1]^+^ = 279.1591; found = 279.1592.

#### 2.1.6. Methyl 4-(4-Allyl-2-Methoxyphenoxy)Butanoate **2d**

Starting from compound **2c** (0.248 g, 1.12 × 10^−3^ mol) and using acetic anhydride (0.16 mL, 1.68 mmol), compound **2d** was obtained as a light yellow oil (0.248 g, 84% yield). *R*_f_ = 0.29 (ethyl acetate/light petroleum 1:10). ^1^H NMR (CDCl_3_, 400 MHz): *δ*_H_ = 2.02 (3H, s, CO_2_C*H_3_*), 2.11 (2H, quint, *J =* 6.8 Hz, OCH_2_CH_2_CH_2_CO_2_CH_3_), 3.30 (2H, d, *J =* 6.8 Hz, CH_2_Ph), 3.81 (3H, s, OC*H_3_*), 4.05 (2H, t, *J =* 6.4 Hz, OC*H_2_*CH_2_CH_2_CO_2_CH_3_), 4.25 (2H, t, *J =* 6.4 Hz, OCH_2_CH_2_CH_2_CO_2_CH_3_), 5.01–5.08 (2H, m, CH=CH_2_), 5.88–5.98 (1H, m, CH=CH_2_), 6.67–6.69 (2H, m, H-3 and H-5), 6.80 (1H, d, *J =* 8.0 Hz, H-6) ppm. ^13^C NMR (CDCl_3_, 100.6 MHz): *δ*_C_ = 20.66 (CO_2_*C*H_3_), 28.43 (OCH_2_*C*H_2_CH_2_CO_2_CH_3_), 39.57 (*C*H_2_Ph), 55.64 (O*C*H_3_), 61.18 (OCH_2_CH_2_*C*H_2_CO_2_CH_3_), 65.60 (O*C*H_2_CH_2_CH_2_CO_2_CH_3_), 112.30 (C-3), 113.53 (C-6), 115.38 (CH=*C*H_2_), 120.26 (C-5), 133.03 (C-4), 137.40 (*C*H=CH_2_), 146.38 (C-1), 149.35 (C-2), 170.76 (*C*O_2_CH_3_) ppm. HRMS: *m/z* (ESI) calculated for C_15_H_20_NaO_4_ [M+Na]^+^ = 287.1254; found = 287.1253.

#### 2.1.7. 4-(4-Allyl-2-Methoxyphenoxy)Butanoic Acid **2f**

To a suspension of compound **2e** (0.200 g, 7.19 × 10^−4^ mol) in 1,4-dioxane (3.0 mL), 1 M aqueous sodium hydroxide (2.16 mL, 2.16× 10^−3^ mol) was added, and compound **2f** was obtained as a white solid (0.158 g, 88 % yield). *R*_f_ = 0.78 (ethyl acetate/light petroleum 1:10). ^1^H NMR (CDCl_3_, 400 MHz): *δ*_H_ = 2.15 (2H, quint, *J =* 7.2 Hz, OCH_2_CH_2_CH_2_CO_2_H), 2.62 (2H, t, *J =* 6.8 Hz, OCH_2_CH_2_CH_2_CO_2_H), 3.34 (2H, d, *J =* 6.4 Hz, CH_2_Ph), 3.85 (3H, s, OCH_3_), 4.06 (2H, t, *J =* 6.0 Hz, OCH_2_CH_2_CH_2_CO_2_H), 5.05 – 5.12 (2H, m, CH=CH_2_), 5.92–6.02 (1H, m, CH=CH_2_), 6.70–6.83 (2H, m, H-3 and H-5), 6.83 (1H, d, *J =* 8.4 Hz, H-6) ppm.^13^C NMR (CDCl_3_, 100.6 MHz): *δ*_C_ = 24.31 (OCH_2_*C*H_2_CH_2_CO_2_H), 30.55 (OCH_2_CH_2_*C*H_2_CO_2_H), 39.77 (*C*H_2_Ph), 55.85 (O*C*H_3_), 67.99 (O*C*H_2_CH_2_CH_2_CO_2_H), 112.45 (C-3), 113.73 (C-6), 115.60 (CH=CH_2_), 120.48 (C-5), 133.26 (C-4), 137.60 (*C*H=CH_2_), 146.49 (C-1), 149.50 (C-2), 179.15 (*C*O_2_H) ppm. HRMS: *m/z* (ESI) calculated for C_14_H_19_O_4_ [M+1]^+^ = 251.1278; found = 251.1274.

#### 2.1.8. 2-(3-Methoxy-4-propoxybenzyl)oxirane **3a**

Starting compound **2a** (0.218 g, 9.70 × 10^−4^ mol) and using *m*-chloroperbenzoic acid (0.434 g, 2.51 × 10^−3^ mol), compound **3a** was obtained as a yellow oil (0.016 g, 7% yield). *R*_f_ = 0.30 (ethyl acetate/light petroleum 1:10). ^1^H NMR (CDCl_3_, 400 MHz): *δ*_H_ = 1.04 (3H, t, *J =* 7.6 Hz, OCH_2_CH_2_CH_3_), 1.82–1.91 (2H, m, OCH_2_CH_2_CH_3_), 2.55 (1H, q, *J =* 2.8 Hz CH_2_ oxirane), 2.76–2.87 (3H, m, CH_2_Ph and CH_2_ oxirane), 3.12–3.17 (1H, m, CH oxirane), 3.86 (3H, s, OCH_3_), 3.97 (2H, t, *J =* 6.8 Hz, OC*H_2_*CH_2_CH_3_), 6.76–6.84 (2H, m, H-2 and H-6), 6.83 (1H, d, *J =* 8.0 Hz, H-5) ppm. ^13^C NMR (CDCl_3_, 100.6 MHz): *δ*_C_ = 10.39 (OCH_2_CH_2_*C*H_3_), 22.47 (OCH_2_*C*H_2_CH_3_), 38.27 (*C*H_2_Ph), 46.78 (*C*H_2_ oxirane), 52.57 (*C*H oxirane) 55.99 (O*C*H_3_), 70.59 (O*C*H_2_CH_2_CH_3_), 112.79 (C-2), 113.13 (C-5), 120.94 (C-6), 129.72 (C-1), 147.36 (C-4), 149.36 (C-3) ppm. HRMS: *m/z* (ESI) calculated for C_13_H_18_NaO_3_ [M+Na]^+^ = 0245.1148; found = 245.1148.

#### 2.1.9. 2-(4-(3-Chloropropoxy)-3-Methoxybenzyl)Oxirane **3b**

Starting from compound **2b** (0.213 g, 8.85 × 10^−4^ mol) and using *m*-chloroperbenzoic acid (0.198 g, 1.15 × 10^−3^ mol), compound **3b** was obtained as a yellow oil (0.151 g, 67% yield). *R*_f_ = 0.52 (ethyl acetate/light petroleum 1:3). ^1^H NMR (CDCl_3_, 400 MHz): *δ*_H_ = 2.28 (2H, quint, *J =* 6.0 Hz, OCH_2_CH_2_CH_2_), 2.55 (1H, q, *J =* 2.8 Hz, CH_2_ oxirane), 2.79–2.83 (3H, m, CH_2_Ph and CH_2_ oxirane), 3.12–3.16 (1H, m, CH oxirane), 3.77 (2H, t, *J =* 6.4 Hz, OCH_2_CH_2_CH_2_), 3.86 (3H, s, OCH_3_), 4.15 (2H, t, *J =* 6.0 Hz, OCH_2_CH_2_CH_2_), 6.77–6.79 (2H, m, H-2 and H-6), 6.86 (1H, d, *J =* 7.6 Hz, H-5) ppm. ^13^C NMR (CDCl_3_, 100.6 MHz): *δ*_C_ = 32.27 (OCH_2_*C*H_2_CH_2_Cl), 38.26 (*C*H_2_Ph), 41.63 (OCH_2_CH_2_*C*H_2_Cl), 46.76 (*C*H_2_ oxirane), 52.53 (*C*H oxirane), 55.93 (O*C*H_3_), 65.82 (O*C*H_2_CH_2_CH_2_Cl), 112.88 (C-6), 113.85 (C-5), 121.01 (C-2), 130.47 (C-1), 146.96 (C-4), 149.54 (C-3) ppm. HRMS: *m/z* (ESI) calculated for C_13_H_17_^35^ClO_3_ [M+Na]^+^ = 279.0758; found = 279.0754; calculated for C_13_H_17_^37^ClO_3_ [M+Na]^+^ = 281.0733; found = 281.0722.

#### 2.1.10. 3-(2-Methoxy-4-(Oxiran-2-Ylmethyl)Phenoxy)Propan-1-ol **3c**

Starting compound **2c** (0.156g, 7.03 × 10^−4^ mol) and using *m*-chloroperbenzoic acid (0.346 g, 2.0 × 10^−3^ mol), compound **3c** was obtained as a yellow oil (0.096 g, 57% yield). *R*_f_ = 0.58 (ethyl acetate). ^1^H NMR (CDCl_3_, 400 MHz): *δ*_H_ = 2.08 (2H, quint, *J =* 6.0 Hz, OCH_2_C*H_2_*CH_2_OH), 2.55 (1H, q, *J =* 2.8 Hz, CH_2_ oxirane), 2.80–2.85 (3H, m, CH_2_Ph and C*H_2_* oxirane), 3.13–3.17 (1H, m, CH oxirane), 3.86 (3H, s, OCH_3_), 3.89 (2H, t, *J =* 5.6 Hz, OCH_2_CH_2_CH_2_OH), 4.19 (2H, t, *J =* 5.6 Hz, OC*H_2_*CH_2_CH_2_OH), 6.71–6.73 (2H, m, H-3 and H-5), 6.84 (1H, d, *J =* 8.4, H-6) ppm. ^13^C NMR (CDCl_3_, 100.6 MHz): *δ*_C_ = 31.75 (OCH_2_*C*H_2_CH_2_OH), 38.34 (*C*H_2_Ph), 46.78 (*C*H_2_ oxirane), 52.56 (*C*H oxirane), 55.83 (O*C*H_3_), 61.57 (OCH_2_CH_2_*C*H_2_OH), 68.64 (O*C*H_2_CH_2_CH_2_OH), 112.50 (C-3), 113.45 (C-6), 120.96 (C-5), 130.52 (C-4), 146.95 (C-1), 149.41 (C-2) ppm. HRMS: *m/z* (ESI) calculated for C_13_H_18_NaO_4_ [M+Na]^+^ = 261.1097; found = 261.1098.

#### 2.1.11. 3-(2-Methoxy-4-(Oxiran-2-Ylmethyl)Phenoxy)Propyl Acetate **3d**

Starting from compound **2d** (0.1039 g, 3.93 × 10^−4^ mol) and using *m*-chloroperbenzoic acid (0.194 g, 1.12 × 10^−3^ mol), compound **3c** was obtained as light yellow oil (0.031g; 28% yield). *R*_f_ = 0.32 (ethyl acetate/light petroleum 1:3). ^1^H NMR (CDCl_3_, 400 MHz): *δ*_H_ = 2.05 (3H, s, CO_2_C*H_3_*), 2.15 (2H, quint, *J =* 6.4 Hz, OCH_2_CH_2_CH_2_CO_2_CH_3_), 2.55 (1H, q *J =* 2.8 Hz,CH_2_ oxirane), 2.79–2.82 (3H, m, CH_2_Ph and C*H_2_* oxirane), 3.12–3.16 (1H, m, CH oxirane), 3.86 (3H, s, OCH_3_), 4.09 (2H, t, *J =* 6.4 Hz, OC*H_2_*CH_2_CH_2_CO_2_CH_3_), 4.28 (2H, t, *J =* 6.4 Hz, OCH_2_CH_2_CH_2_CO_2_CH_3_), 6.77–6.79 (2H, m, H-3 and H-5), 6.84 (1H, d, *J =* 8.0 Hz, H-6) ppm. ^13^C NMR (CDCl_3_, 100.6 MHz): *δ*_C_ = 20.90 (CO_2_*C*H_3_), 23.79 (OCH_2_*C*H_2_CH_2_CO_2_CH_3_), 38.26 (*C*H_2_Ph), 46.77 (CH*_2_* oxirane), 52.55 (*C*H oxirane), 55.93 (O*C*H_3_), 61.37 (OCH_2_CH_2_*C*H_2_CO_2_CH_3_), 65.73 (O*C*H_2_CH_2_CH_2_CO_2_CH_3_), 112.88 (C-3), 113.59 (C-6), 120.96 (C-5), 130.33 (C-4), 147.02 (C-1), 149.51 (C-2), 170.07 (*C*O_2_CH_3_) ppm. HRMS: *m/z* (ESI) calculated for C_15_H_20_NaO_5_ [M+Na]^+^ = 303.1203; found = 303.1202.

#### 2.1.12. Ethyl 4-(2-Methoxy-4-(Oxiran-2-Ylmethyl)Phenoxy)Butanoate **3e**

Starting from compound **2e** (0.173 g, 6.22 × 10^−4^ mol) and *m*-chloroperbenzoic acid (0.278 g, 1.61 × 10^−3^ mol), compound **3e** was obtained as a yellow oil (0.022 g, 13% yield). *R*_f_ = 0.31 (ethyl acetate/light petroleum 1:3). ^1^H NMR (CDCl_3_, 400 MHz): *δ*_H_ = 1.26 (3H, t, *J =* 7.2 Hz, CO_2_CH_2_C*H_3_*), 2.14 (2H, quint, *J =* 7.2 Hz, OCH_2_C*H_2_*CH_2_CO_2_CH_2_CH_3_), 2.51–2.56 (3H, m, C*H_2_* oxirane and OCH_2_CH_2_CH_2_CO_2_CH_2_CH_3_), 2.76–2.86 (3H, m, CH_2_Ph and CH_2_ oxirane), 3.12–3.16 (1H, m, CH oxirane), 3.86 (3H, s, OCH_3_), 4.05 (2H, t, *J =* 6.0 Hz, OCH_2_CH_2_CH_2_CO_2_CH_2_CH_3_), 4.14 (2H, q, *J =* 7.2 Hz CO_2_CH_2_CH_3_), 6.75–6.79 (2H, m, H-3 and H-5), 6.84 (1H, d, *J =* 8.0 Hz, H-6) ppm. ^13^C NMR (CDCl_3_, 100.6 MHz): *δ*_C_ = 14.19 (CO_2_CH_2_*C*H_3_), 24.57 (OCH_2_*C*H_2_CH_2_CO_2_CH_2_CH_3_), 30.74 (OCH_2_CH_2_*C*H_2_CO_2_CH_2_CH_3_), 38.29 (CH_2_Ph), 46.78 (CH_2_ oxirane), 52.55 (CH oxirane), 55.95 (O*C*H_3_), 60.36 (CO_2_*C*H_2_CH_3_), 68.07 (O*C*H_2_CH_2_CH_2_CO_2_CH_2_CH_3_), 112.87 (C-3), 113.58 (C-6), 120.98 (C-5), 130.18 (C-4), 147.09 (C-1), 149.50 (C-2), 173.20 (*C*O_2_CH_2_CH_3_) ppm. HRMS: *m/z* (ESI) alculated for C_15_H_22_NaO_5_ [M+Na]^+^ = 317.1359; found = 317.1359.

### 2.2. Screening of Toxicity Towards Insect Cells

**Figure 1:**
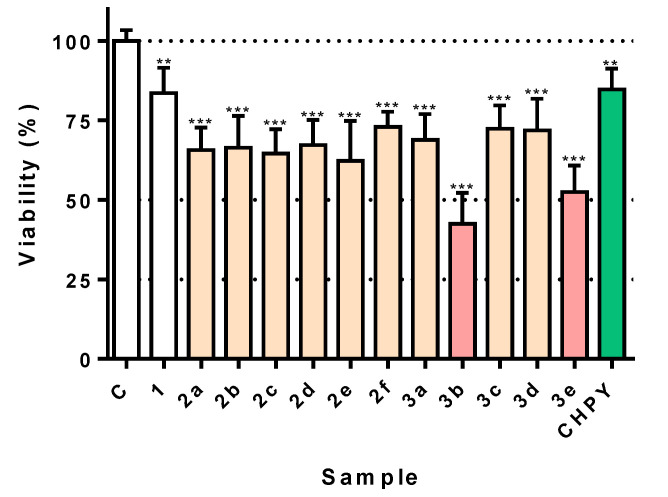
Viability of cells exposed to the molecules under study **2a–f** and **3a–e** (100 µg/mL), medium (control), or the reference insecticide chlorpyrifos (CHPY; 100 µg/mL). Cells were incubated for 24 h, after which viability was evaluated. ** *p* < 0.01; *** *p* < 0.001.

As a model for insecticide activity, two-dimensional (2D) cultures of *Sf9* cells, which derive from the ovary cells of *Spodoptera frugiperda* (a common pest) were used. All molecules were assayed at the same concentration (100 µg/mL) in order to allow direct comparison of their potency. The starting material, eugenol **1**, was nearly devoid of any toxicity, causing a marginal decrease of viability (Figure 1). All eugenol derivatives arising from alkylation reactions of the hydroxyl group, and possessing a propyl chain with hydrogen, hydroxyl, ester, chlorine, and carboxylic acid as terminals (**2a–f**) displayed higher toxicity than the starting eugenol **1**, with cells showing around 55–65% of viability (Figure 1). When analyzing the results of the oxiranes **3a–e** series, which results from the epoxidation of the compound **2** series, a distinct trend was found. In a general way, all members of the 3 series displayed enhanced toxicity when compared to 1. Among all derivatives, **3b** and **3e** were clearly the most potent, with the latter eliciting ca. 50% viability loss, while the former reached nearly 60% viability loss, nearly double the effect of the commercial insecticide chlorpyrifos (Figure 1). For this reason, these two molecules were further characterized for their effect.

### 2.3. Impact of Eugenol Derivatives ***3b*** and ***3e*** in Insect Cell Morphology

We were interested in assessing the impact that **3b** and **3e** had in the morphology of the insect cells under study. For this reason, treated cells were imaged to evaluate chromatin status and overall cell morphology. We used both molecules at the same concentration that had been assessed for viability studies (100 µg/mL); however, the effect of **3e** was so pronounced that no cells remained. For this reason, in the specific case of this molecule, the concentration of 50 µg/mL was used.

Incubation with either molecules resulted in reduced cell density when compared with control cells, which is in line with the results reported for their impact in cell viability.

**Figure 2:**
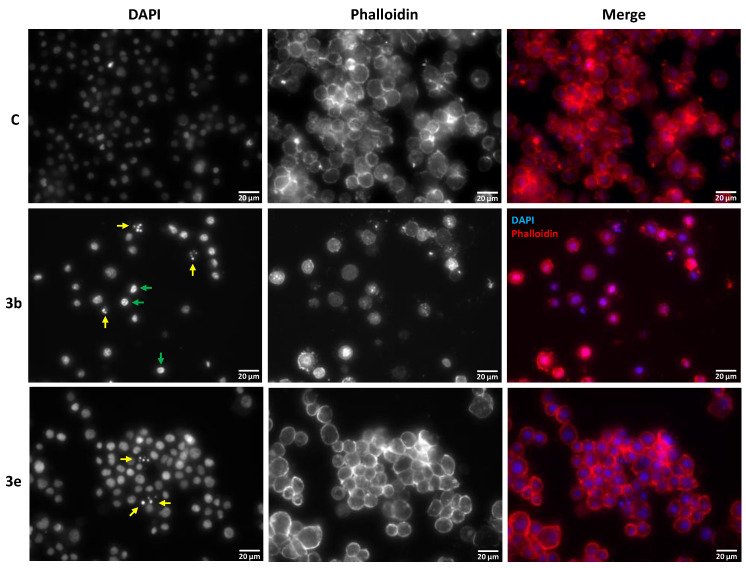
Morphology of *Sf9* cells exposed to compounds **3b** (100 µg/mL) and **3e** (50 µg/mL) after 24 h of incubation (S Plan Fluor ELWD 40× DIC N1 objective). Overall cell morphology was evaluated using phalloidin (actin) and DAPI (chromatin status). Yellow arrow: chromatin fragmentation; green arrow: chromatin condensation; C: control. Color blind-friendly image in Supplementary Figure S1.

When examining the 4’, 6-diamidino-2-phenylindole (DAPI) channel (Figure 2), it was clear that the **3b**- and **3e**-treated cells exhibited chromatin changes, karyorrhexis (nuclear fragmentation; yellow arrows), and pyknosis (chromatin condensation; green arrows). We have quantified this effect (Supplementary Table S1), results, showing that incubation with **3b** resulted in an increase of fragmented and condensed chromatin to 34.8 ± 0.3% and 12.6 ± 4.2%, respectively, while **3e** elicited 21.2 ± 0.8% and 5.8 ± 0.5%, respectively.

### 2.4. Oxiran-Bearing Eugenol Derivatives ***3b*** and ***3e*** Activate Caspase-Like Proteases in Sf9 Cells

**Figure 3:**
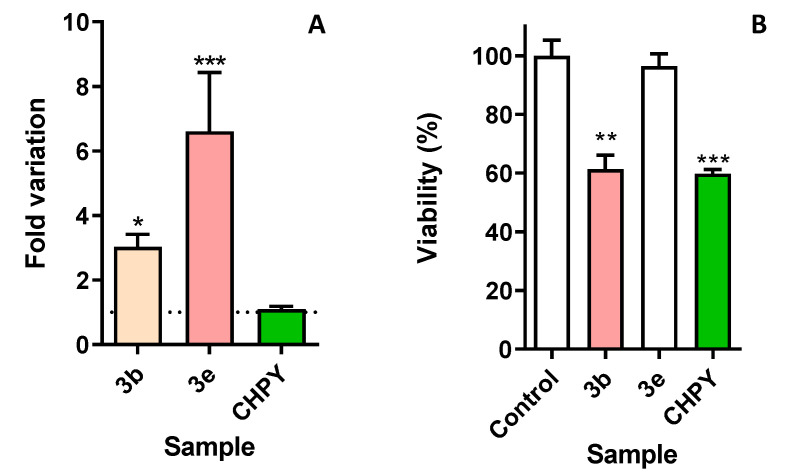
(**A**) Caspase-like activity found in *Sf9* cells after incubation with compounds **3b** (100 µg/mL) and **3e** (50 µg/mL), or the reference insecticide chlorpyrifos (CHPY, 100 µg/mL), for 24 h. (**B**) Viability of human keratinocytes exposed to compounds **3b** and **3e** (100 µg/mL), medium (control), or the reference insecticide chlorpyrifos (CHPY, 100 µg/mL). Cells were incubated for 24 h, after which viability was evaluated. * *p* < 0.05; ** *p* < 0.01; *** *p* < 0.001.

Considering the lack of commercial options for the assessment of insect counterparts of mammalian caspases, it was hypothesized that the degree of homology described between insect and mammalian caspases [36] should be high enough for substrate cross-reactivity. For this reason, a substrate for mammalian executor caspase isoforms 3/7 has been used. As shown in Figure 3A, incubation of cells with **3b** resulted in an about three-fold increase in caspase-like activity, reaching over six-fold in the case of **3e**. For benchmarking purposes, the commercial insecticide chlorpyrifos was also evaluated, and its effect was not statistically different from control cells.

### 2.5. Some Eugenol Derivatives are Selectively Toxic to Insect but Not Human Cells

Bearing in mind the importance of developing new and more potent insecticides that have a safe toxicological profile towards mammalian organisms, the impact of **3b** and **3e** in human cells has been evaluated. Considering the usual routes of poisoning, specifically skin, human keratinocytes, were chosen. As it can be seen in Figure 3B, **3b** caused around 40% loss of cell viability (equivalent to the commercial insecticide chlorpyrifos), which is still less pronounced than the effect elicited in insect cells. In the case of **3e**, Figure 3B shows that it had no effect in human cells at the same concentration in which it caused around 50% of cell viability loss in insect cells, thus displaying a selective effect towards the latter.

## 3. Discussion

### 3.1. Synthesis of Eugenol Derivatives ***2a*–*f*** and ***3a*–*e***

Structural modifications in the hydroxyl group and double bond of 4-allyl-2-methoxyphenol, eugenol **1**, obtained by hydrodistillation of clove, were carried out. Alkylation of the hydroxyl group of 4-allyl-2-methoxyphenol **1** with 1-bromopropane,1-bromo-3-chloropropane, 3-bromopropan-1-ol, and ethyl 4-bromobutanoate, using cesium carbonate as a base and by heating at 65 °C in acetonitrile, gave 4-allyl-2-methoxy-1-propoxybenzene **2a**, 4-allyl-1-(3-chloropropoxy)-2-methoxybenzene **2b**, 3-(4-allyl-2-methoxyphenoxy)propan-1-ol **2c**, and ethyl 4-(4-allyl-2-methoxyphenoxy)butanoate **2e**, respectively.

Compound **2c** was further reacted with acetic anhydride by heating at 65 °C to obtain 3-(4-allyl-2-methoxyphenoxy)propyl acetate **2d**. In addition, compound **2e** was subjected to hydrolysis with aqueous 1M NaOH in 1,4-dioxane at room temperature to give 4-(4-allyl-2-methoxyphenoxy)butanoic acid **2f**. Compounds **2a**–**e** were obtained as oils or a solid material (**2f**) in 53% to 88% yields, and were fully characterized by ^1^H and ^13^C NMR spectroscopy, as well as HRMS. The ^1^H NMR spectra of compounds **2a**–**f** showed the different characteristic signals for the aliphatic protons of methylene and methyl groups (*δ =* 1.03–4.30 ppm), as well as the expected protons for the eugenol’s double bond as multiplets, CH_2_ (*δ* = 5.01–5.14 ppm) and CH (*δ* = 5.90–6.04 ppm). ^13^C NMR spectra of all compounds showed signals of the aliphatic carbons from the methylene and methyl groups (*δ* = 10.23–70.44 ppm), and for compounds **2d**, **2e**, and **2f** was visible the presence of signals for the carbonyl groups (*δ* = 170.76–179.15 ppm).

To perform epoxidation of the double bond of eugenol derivatives, compounds **2a**–**e** reacted with *m*-chloroperbenzoic acid in dichloromethane at room temperature, and the respective derivatives, namely 2-(3-methoxy-4-propoxybenzyl)oxirane **3a**, 2-(4-(3-chloropropoxy)-3-methoxybenzyl)oxirane **3b**, 3-(2-methoxy-4-(oxiran-2-ylmethyl)phenoxy)propan-1-ol **3c**, 3-(2-methoxy-4-(oxiran-2-ylmethyl)phenoxy)propyl acetate **3d**, and ethyl 4-(2-methoxy-4-(oxiran-2-ylmethyl)phenoxy)butanoate **3e** were obtained. These compounds **3a**–**e** were isolated as yellow oils in yields up to 67%, and were fully characterized by the usual analytical techniques. It stands out that epoxidation of compounds **2a**–**e** was verified by the presence of the protons signals related the oxirane ring (*δ* = 2.52–3.17 ppm) and the absence of the signals of protons for the double bond of eugenol skeleton. The presence of carbon signals relative to oxirane ring, CH_2_ (*δ* = 46.76–46.78 ppm, and CH = (*δ* 52.53–52.57 ppm) also confirmed the structure of expected eugenol derivatives **3a–e**.

### 3.2. Differential Effect of Eugenol Derivatives Towards Insect Cells

When evaluating the impact of all molecules obtained, a clear trend could be established. All derivatives arising from alkylation reactions of the hydroxyl group and possessing a propyl chain with hydrogen, hydroxyl, ester, chlorine, and carboxylic acid as terminals (**2a–f**) displayed higher toxicity than the starting molecule **1** (Figure 1). In a general way, all member of the 3 series displayed enhanced toxicity when compared to eugenol, which suggests a role for the oxirane group in the potency of this family of compounds. Among all derivatives, **3b** and **3e** were clearly the most potent, eliciting a degree of viability loss higher than the commercial insecticide chlorpyrifos (Figure 1).

### 3.3. Eugenol Derivatives Trigger a Process of Programmed Cell Death

The results from morphological assessment showed the advent of chromatin changes, such as karyorrhexis and pyknosis (Figure 2). This result, although not conclusive per se, was compatible with a process of programmed cell death. For this reason, we decided to assess the activity of caspase homologues, given the role and relevance of these serine proteases in several types of programmed cell deaths. As shown in Figure 3A, incubation of cells with **3b** resulted in an about three-fold increase in caspase-like activity; this value increased to over six-fold in the case of **3e**. For benchmarking purposes, the commercial insecticide chlorpyrifos was also evaluated, and its effect was not statistically different from control cells. This result is not completely unexpected, considering that chlorpyrifos belongs to the class of organophosphates pesticides, thus exerting its toxicity by disrupting the nervous system of the target organisms, specifically by irreversibly inhibiting acetylcholinesterase, leading to the build-up of acetylcholine levels [37]. Although death can ultimately occur in vivo, apoptosis is not the primary result of its effect. The fact that the eugenol derivatives described herein exert their effect via a distinct mechanism than organophosphates, which are known for their toxicity, is promising. Furthermore, in view of the chemical structures of the molecules presented here, eugenol derivatives are unlikely to inhibit acetylcholinesterase, which suggests that they may act through distinct mechanisms.

### 3.4. New Eugenol Derivatives are Not Toxic to Human Cells

Finally, the effect of the most promising molecules, **3b** and **3e**, was evaluated towards human cells. Considering the most frequent routes of contact with pesticides, we have chosen keratinocytes as a model, given their pivotal role in skin anatomy. As shown in Figure 3B, very distinct selectivity was found. Compound **3b** caused around 40% loss of cell viability, which is still less than the effect elicited in insect cells. In the case of **3e**, Figure 3B shows that it had no effect in human cells at the same concentration in which it caused around 50% of cell viability loss, thus displaying a selective effect towards insect cells. Even more interesting is that this selective effect outperforms that of the commercial insecticide chlorpyrifos, which was highly toxic to keratinocytes and caused over 40% of viability loss.

## 4. Materials and Methods

### 4.1. Chemicals

Dichloromethane, acetonitrile, ethyl acetate, light petroleum, 1,4-dioxane, 1-bromo-3-chloropropane, cesium carbonate, and *m*-chloroperbenzoic acid were purchased from Fisher Scientific (Geel, Belgium). The 1-Bromopropane, 3-bromopropan-1-ol and ethyl 4-bromobutanoate were from Sigma-Aldrich (St. Louis, MO, United States). The anhydrous magnesium sulphate and acetic anhydride were PanReac Applichem (Barcelona, Spain) products. Chloroform-*d* was produced by Eurisotop (Cambridge, England). Thin-layer chromatography (TLC) analyses were carried out on 0.25 mm-thick, precoated silica plates (Merck Fertigplatten Kieselgel 60F254, Germany), and spots were visualized under UV light. Chromatography on silica gel was carried out on Merck Kieselgel (230–240 mesh).

### 4.2. Analytical Instruments 

The NMR spectra were obtained on a Bruker Avance III at an operating frequency of 400.0 MHz for ^1^H NMR and 100.6 MHz for ^13^C NMR, using the solvent peak as internal reference at 25 °C. All chemical shifts are given in ppm using *δ* Me_4_Si = 0 ppm as reference, and *J* values are given in hertz. Assignments were made by a comparison of chemical shifts, peak multiplicities, and *J* values, and were supported by spin decoupling–double resonance and bidimensional heteronuclear correlation techniques. High-resolution mass spectrometry analyses were performed at the CACTUS, Unidade de Masas e Proteómica, at the University of Santiago de Compostela, Spain.

### 4.3. Synthesis of Eugenol Derivatives ***2a*–*f*** and ***3a*–*e***

#### 4.3.1. Extraction of Eugenol **1** from *Syzygium Aromaticum*

The extraction of 4-allyl-2-methoxyphenol, eugenol **1**, was made from *Syzygium aromaticum* (cloves) in a round-bottom flask containing distilled water (200 mL) and the cloves (21.415 g). Hydrodistillation assembly was performed, and the mixture was refluxed during 2 h. The distillate was extracted with dichloromethane (3 × 150 mL), the organic phase was dried over anhydrous magnesium sulphate, and solvent evaporation under vacuum yielded 4-allyl-2-methoxyphenol, eugenol (**1**).

#### 4.3.2. General Procedure for Synthesizing Compounds **2a**–**c** and **2e**

To a solution of 4-allyl-2-methoxyphenol **1** (1 equiv) in acetonitrile (5 mL), the corresponding alkyl halide (1.1 equiv) and cesium carbonate (5 equiv) were added, and the resulting mixture was heated at 60 °C for 2 h 30 min. The progress of the reaction was monitored by TLC (ethyl acetate/light petroleum = 1:10). The excess of base was filtered, the solvent was evaporated, and the crude mixture was purified by column chromatography on silica gel using ethyl acetate/light petroleum (1:10) as the eluent.

#### 4.3.3. General Procedure for Synthesizing Compound **2d**

To compound **2c** acetic anhydride was added, and the resulting mixture was stirred at 65 °C for 12 h. The reaction was monitored by TLC (ethyl acetate/light petroleum = 1:10). After completion, the mixture was diluted with ethyl acetate and washed with sodium bicarbonate (2 × 5 mL); the organic phase was dried with anhydrous magnesium sulfate, and the solvent was evaporated.

#### 4.3.4. General Procedure for Synthesizing Compound **2f**

To a suspension of compound **2e** in 1,4-dioxane, 1 M aqueous sodium hydroxide was added. The solution was stirred at room temperature for 8 h, and acidified to pH 2–3 with 1 M aqueous potassium hydrogen sulfate. The reaction mixture was evaporated and dichloromethane was added, giving a precipitate that was filtered. Then the solvent was evaporated.

#### 4.3.5. General Procedure for Synthesizing Compounds **3a**–**e**

The respective precursor (1 equiv) **2a–e** (4 mL) dissolved in dichloromethane was added dropwise to a solution of *m-*chloroperbenzoic acid (1 equiv) in dichloromethane (6 mL) at 0 °C. After stirring for 1 h, *m*-chloroperbenzoic acid was again added (1 equiv), and the reaction mixture was stirred for more 12 h. A 10% aqueous solution of sodium sulfate (10 mL) was added, and the resulting solution was washed with 5% aqueous solution of sodium hydrogen carbonate (2 × 10 mL). The organic phase was dried with anhydrous magnesium sulfate, and the solvent was evaporated.

### 4.4. Preparation Methods

#### 4.4.1. Cell Culture

*Sf9* (*Spodoptera frugiperda*) cells were maintained as a suspension culture and cultivated in Grace’s medium with 10% FBS and 1% penicillin/streptomycin, at 28 °C. Cells were used in experiments while in the exponential phase of growth. On the other hand, HaCaT (human keratinocyte) cells were cultured in Dulbecco’s modified eagle medium (DMEM) supplemented with 10% fetal bovine serum (FBS) and 1% penicillin/streptomycin at 37 °C, in a humidified atmosphere of 5% CO_2_.

#### 4.4.2. Viability Assessment

For the assessment of viability, a resazurin-based method was used. *Sf9* and HaCaT cells were plated at a density of 3.0 × 10^4^ and 1.5 × 10^4^ cells/well, respectively, incubated for 24 h, and then exposed to the molecules under study for 24 h. After this period, a commercial solution of resazurin was added (1:10), and the kinetic reaction of fluorescence increase monitored at 560/590 nm. For HaCaT and *Sf9* cells, 30 and 60 min of incubation were used, respectively.

#### 4.4.3. Morphological Assessment

For morphological studies, *Sf9* cells were cultured in 96-well plates at the same density used for viability experiments, in the presence of the molecules under study. After incubation, cells were washed with Hanks’ balanced salt solution (HBSS) and fixed in 10% formalin solution for 30 min, at room temperature. CF543 (5 U/mL) and DAPI (0.25 µg/mL) were added, and cells were stained for 25 min at room temperature and washed with HBSS.

Images were acquired in an inverted Eclipse Ts2R-FL (Nikon) equipped with a Retiga R1 camera and an S Plan Fluor ELWD 20x DIC N1 objective. Images were analyzed with Fiji [38]. For quantitative parameters, the Fiji’s Cell Counter plugin was used.

#### 4.4.4. Caspase-Like Activity

*Sf9* cells were plated at the same density described for viability studies and exposed to the molecules under study for the designated time. Generally, the same method described by the authors previously for mammalian cells [39] was used; however, it was adapted for insect cells. After the incubation period, caspase-3/7 substrate was added to wells and cells incubated for 20 min at 22 °C. The luminescent signal was measured in a microplate reader (Cytation 3, BioTek, Winooski, VT, USA), and was performed in duplicate in three independent experiments.

#### 4.4.5. Statistical Analysis

For biological assays, the Shapiro–Wilks normality test was performed in the data to ensure that it followed a normal distribution. Comparison between the means of controls and each experimental condition was performed using ANOVA. Outliers were identified by the Grubbs’ test. Data was expressed as the mean ± standard deviation (SD) of at least three independent experiments. GraphPad Prism 7.0 software was used, and values were considered statistically significant when *p* < 0.05.

## 5. Conclusions

Overall, with the present work it was demonstrated that medicinal chemistry approaches are valid strategies for obtaining new semisynthetic derivatives of a natural essential oil, which can act as promising alternatives to the available synthetic insecticides. Having started with a natural molecule devoid of activity, eugenol **1**, it was possible to obtain several molecules with enhanced activity, some of them highly potent at concentrations as low as 100 μg/mL. The most potent molecules were shown to elicit morphological changes compatible with some processes of programmed cell death, being also capable of increasing the activity of serine proteases pivotal to some of these death pathways, notably caspases.

Finally, it was showed that ethyl 4-(2-methoxy-4-(oxiran-2-ylmethyl)phenoxy)butanoate **3e** exhibits a more favorable safety profile towards human cells than that of the commercial insecticide chlorpyrifos, thus paving the way for the design of new alternatives to insecticides currently in use.

## Supplementary Materials

The following are available online at https://www.mdpi.com/1422-0067/21/23/9257/s1.

## Abbreviations

EOs	Essential oils
TLC	Thin-layer chromatography
NMR	Nuclear magnetic resonance
HRMS	High-resolution mass spectrometry
Sf9	Spodoptera frugiperda
