# Supplementary material for: New Eugenol Derivatives with Enhanced Insecticidal Activity
Source: Int J Mol Sci. 2020 Dec 4;21(23):9257. doi: 10.3390/ijms21239257 (PMC7729565; doi:10.3390/ijms21239257)
Supplement: Supplementary file 1 [file ijms-21-09257-s001.pdf]

Supplementary Information

New Eugenol Derivatives with Enhanced Insecticidal Activity

Maria José G. Fernandes [1], Renato B. Pereira [2], David M. Pereira [2,*], A. Gil Fortes [1], Elisabete M. S. Castanheira [3] and M. Sameiro T. Gonçalves [1,*]

1 Centre of Chemistry, Department of Chemistry, Campus of Gualtar, University of Minho, 4710-057 Braga, Portugal; mjfernandes@quimica.uminho.pt (M.J.G.F.); gilf@quimica.uminho.pt (A.G.F.)

2 REQUIMTE/LAQV, Laboratory of Pharmacognosy, Department of Chemistry, Faculty of Pharmacy, University of Porto, R. Jorge Viterbo Ferreira, 228, 4050-313 Porto, Portugal; rjpereira@ff.up.pt

3 Centre of Physics, Department of Physics, Campus of Gualtar, University of Minho, 4710-057 Braga, Portugal; ecoutinho@fisica.uminho.pt

* Correspondence: dpereira@ff.up.pt (D.M.P.); msameiro@quimica.uminho.pt (M.S.T.G.); Tel.: +351-253-604-372 (M.S.T.G.)

Received: 2 November 2020; Accepted: 1 December 2020; Published: date

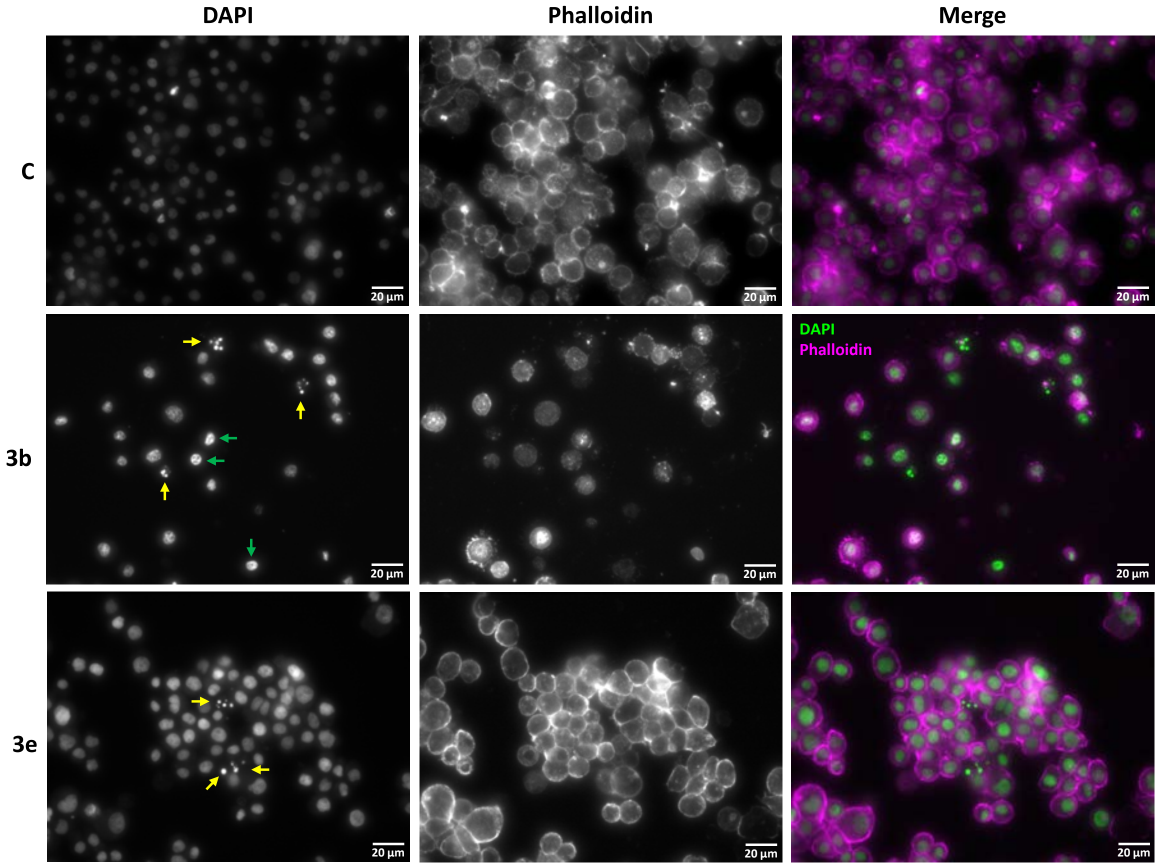

Figure S1 - Morphology of *Sf*9 cells exposed to the indicated molecules (3b 100 µg mL^{-1}; 3e 50 µg mL^{-1}) after 24 h of incubation (S Plan Fluor ELWD 40x DIC N1 objective). Overall cell morphology was evaluated using phalloidin (actin) and chromatin status (DAPI).Yellow arrow: chromatin fragmentation; Green arrow: chromatin condensation. C – Control.

Table S1 – Percentage of cells displaying normal, condensed and fragmented chromatin in control, **3b**- and **3e**-treated *Sf*9 cells. Conditions as in **Figure 2**.

	**Normal**	**Condensed**	**Fragmented**
**C**	95.5 ± 1.8 %	4.3 ± 1.9 %	-
**3b**	52.7 ± 4.2%	34.8 ± 0.3 %	12.6 ± 4.2 %
**3e**	73.0 ± 0.8 %	21.2 ± 0.8 %	5.8 ± 0.5 %
